# Glossary

**Published:** 1997

Acetaldehyde: The immediate product of alcohol *oxidation*, this compound can damage the cellular microstructure of the liver, induce *fibrosis*, affect energy metabolism, and generate *free radicals*.
Acid-base balance: Normal body fluid pH (i.e., hydrogen ion concentration), which must be narrowly regulated for proper body functioning.
Acidosis: A condition in which body fluids become too acidic (see *alkalosis*).
Active transport: The transfer of substances (i.e., molecules or *ions*) across a cell membrane from a lower to a higher concentration, thus requiring energy expenditure.
Alcohol dehydrogenase: An *enzyme* that breaks down alcohol.
Alkalosis: A condition in which body fluids become too alkaline (see *acidosis*).
Allele: One of two or more variants of a *gene*. Different alleles for a gene serve the same function (e.g., code for a *protein* that affects a person’s eye color) but may result in different *phenotypes* (e.g., blue eyes or brown eyes).
Amino acids: The building blocks of *proteins*. Some amino acids also function as *neurotransmitters*.
Ammonia: A neurotoxic chemical compound that is formed in the body primarily as a product of *protein* metabolism.
Anemia: A blood condition in which the number of functional red blood cells is below normal.
Anterior pituitary: A small gland at the base of the brain that is controlled by the *hypothalamus* and which manufactures hormones influencing many organs in the body.
Antibody: A *protein* that is produced by *B cells* in response to, and which interacts with, an *antigen*.
Antidiuretic hormone (ADH): A hormone produced in the *hypothalamus* and released from the posterior pituitary gland in response to dehydration; plays an important role in regulating fluid excretion.
Antigen: Any substance that is recognized by *B cells* or *T cells* and stimulates them to initiate an immune response.
Antioxidants: Chemicals (e.g., glutathione and vitamins A and E) that prevent certain destructive chemical processes in cells.
Apoptosis: A series of chemical reactions within a cell that are induced by various events and which result in the cell’s death.
Atherogenesis: The development of *atherosclerosis*.
Atherosclerosis: A disease of the arteries in which fatty plaques accumulate on the arteries’ inner walls, usually leading to narrowing and “hardening” of the arteries and eventually obstructing blood flow.
Atrial fibrillation: A loss of coordinated contraction that occurs in one or both of the upper chambers of the heart, resulting in rapid and irregular heart and pulse rates.
Atrophy: Wasting away, or shrinkage, of tissue; caused by cell death rather than shrinkage of individual cells.
B lymphocyte (B cell): A type of *white blood cell* that originates in the bone marrow and is distributed throughout the blood and *lymphoid tissues*. B cells produce *antibodies* when stimulated by the appropriate *antigens*.
Basal ganglia: A group of nerve cell structures at the base of the brain that are involved in motor control.
Catalyst: Any substance that facilitates a chemical reaction and which does not undergo a permanent chemical change itself.
Catecholamines: A group of physiologically active substances with various roles in the functioning of the sympathetic and central nervous systems.
Cell-mediated immune response: An immune response provided by the direct actions of immune system cells (primarily *T cells*), as opposed to an immune response mediated by *antibodies* (i.e., *humoral immune response*).
Cellular toxin: A toxin that is released from a cell; also called *endotoxin*.
Cerebellum: The brain structure at the base of the brain that is involved in the control of muscle tone, balance, and sensorimotor coordination.
Cerebral cortex: The intricately folded outer layer of the brain, composed of nerve-cell bodies and gray matter, that covers the *cerebrum*. The cerebral cortex contains areas for processing sensory information and for controlling motor functions, speech, higher cognitive functions, emotions, behavior, and memory.
Cerebrum: The largest portion of the brain; includes the cerebral hemispheres (see *cerebral cortex* and *basal ganglia*).
Chemokines: Small *proteins* secreted by immune cells that can attract other immune cells to the tissue site where the chemokines are produced. Chemokines play a role in *chemotaxis*.
Chemotaxis: The directed movement of a cell in response to a stimulus, such as a *chemokine*.
Cholesterol: A fatlike substance that is an important component of cell membranes and is the precursor of many steroid hormones and bile salts. High cholesterol levels are associated with coronary artery disease.
Cholesteryl ester: The product of a reaction between *cholesterol* and an organic acid.
Cholesteryl ester transfer protein (CETP): A compound that transports *cholesteryl esters* from high density lipoproteins to *low density lipoproteins* for eventual removal from the blood.
Complement: A group of *proteins* circulating in the blood that are either bound to *antigen* and activated by *antibodies* or are activated by molecules found on the surface of some bacteria. Complement activation results in the attraction of *phagocytes*, the release of chemicals that amplify immune responses, and the destruction of invading bacteria.
Cortisol: A *glucocorticoid* produced by the adrenal gland that helps regulate metabolism.
Cytochrome: An *enzyme* that detoxifies foreign compounds.
Cytokine: A molecule that regulates cellular interaction and cellular functions. Cytokines are produced and secreted by a variety of cells, including immune cells.
Diencephalon: The area of the brain consisting of the *thalamus*, which is the brain’s relay center to the *cerebral cortex*, and the *hypothalamus*.
Differentiation: A developmental process during which cells become increasingly specialized and acquire new characteristics and functions.
Diuretic: An agent that increases urine production.
Diuresis: Increased urine production.
DNA: The abbreviation for deoxyribonucleic acid, a molecular component of chromosomes that encodes the genetic information in all organisms except some viruses. DNA molecules usually consist of two strings of *nucleotides*.
Down-regulation: A decrease in the number or sensitivity of *receptors* as a regulatory mechanism to compensate for increased activation of the receptors.
Eicosanoids: The physiologically active substances derived from arachidonic acid (i.e., the prostaglandins, *leukotrienes*, and *thromboxanes*).
Electrolyte: A substance that breaks down into electrically charged *ions* when dissolved in solution. Electrolytes are essential to physiological functioning.
Embolism: The obstruction of a blood vessel by a blood clot or other substance (e.g., a fat droplet) that has been transported through the bloodstream from another part of the body.
Endothelin: An extremely potent vasoconstrictor (i.e., agent that causes narrowing of the blood vessels).
Endotoxin: A potent toxin contained in the cell walls of bacteria found in the intestine; it is released when a bacterium dies and is broken down.
Enzyme: A *protein* that directs and accelerates (i.e., catalyzes) chemical reactions in the body, such as the breakdown of complex molecules into simpler ones, but does not itself undergo permanent change.
Epithelium: The cell layer(s) covering the body’s organs and lining the vessels, body cavities, glands, and organs. The epithelia of different organs consist of different types of cells.
Extracellular fluid: All fluids outside the cells, including the noncellular portion of blood (i.e., plasma).
Ferritin: An iron-containing compound that regulates the storage and transport of iron in the cells.
Fibrin: An insoluble *protein* that forms the basis of a blood clot by linking with similar molecules in a fibrous meshwork. It is the ultimate product in the process of coagulation.
Fibrinolysis: Dissolution of a blood clot through digestion of *fibrin* by *plasmin*.
Fibrosis: Formation of scar tissue.
Folic acid: A vitamin of the B group that is essential for cell growth, cell division, and the absorption of nutrients from the intestines.
Free radicals: Highly reactive molecules that are incapable of existing in a free state for a prolonged period.
Gene: A string of *nucleotides* that directs the synthesis of a *protein*.
Genotype: The genetic makeup of an individual organism.
Glomerulus: A tiny ball or tuft of capillaries projecting into the capsule at the “head” of each *nephron* tubule.
Glucocorticoid: See *cortisol*.
Hematopoiesis: The production and development of all blood cells.
Hemodynamics: The forces and mechanics involved in blood circulation (as through the kidney or another body part).
Hemoglobin: The oxygen-carrying molecule in red blood cells.
Hemolysis: The destruction of red blood cells and the associated release of *hemoglobin*.
Hepatic encephalopathy: Portal-systemic encephalopathy (PSE); a progressive metabolic liver disorder that affects intellectual functioning.
HMG-CoA (Hydroxymethylglutaryl coenzyme A) reductase: An *enzyme* that plays a role in the biosynthesis of *cholesterol*.
Humoral immune response: An immune response provided by *antibodies* circulating in the body’s fluids, primarily the blood and lymph, as opposed to an immune response provided by the direct actions of immune cells (see *cell-mediated immune response*).
Hypertension: High blood pressure.
Hypothalamus: A region of the brain that is involved with basic behavior and physiological functions.
Hypoxia: Below-normal levels of oxygen in inspired gases, arterial blood, or tissue.
Inflammatory response: Redness, swelling, heat, and pain produced in response to tissue injury or infection as the result of increased blood flow and an influx of *white blood cells* and *cytokines* to the affected site.
IGF-1: A *protein* produced by the liver in response to growth hormone (GH) that carries out some of the effects of GH at tissue level.
Interleukin: A group of *cytokines* with various immune system functions.
Interstitial fluid: Fluid between cells.
Intracellular fluid: Fluid within cells.
Ion: An electrically charged atom or group of atoms.
Isoenzymes: Variants of one *enzyme* that perform the same function but may have different properties.
Jaundice: A yellowish staining of the skin, whites of the eyes, and deeper tissues produced by an accumulation of metabolic end products in the blood; often a symptom of liver disease.
Kupffer cells: Specialized immune cells in the liver that filter bacteria and other foreign organic substances from the blood.
Lecithin-cholesterol acyl transferase (LCAT): An *enzyme* that transforms *cholesterol* to *cholesteryl esters*.
Lesion: A wound, injury, or pathological change in a body tissue.
Leukocytes: See *white blood cells*.
Leukocytosis: A blood condition in which the number of *white blood cells* is higher than normal.
Leukotrienes: A class of biologically active compounds that occur in *white blood cells* and induce allergic and *inflammatory* reactions.
Lipids: A family of complex molecules, including fats and fatlike molecules (e.g., fatty acids, steroids, or glycerides) that, among other functions, serve as an energy source and constitute part of the cell membrane.
Lipid peroxidation: The destructive metabolism of fatty substances in cells.
Lipopolysaccharide: A compound or complex of *lipids* and carbohydrates.
Lipoproteins: Complexes consisting of *proteins* and fats or other *lipids* that are important for transporting lipids throughout the body.
Lipoprotein lipase: An *enzyme* involved in the breakdown of substances such as *very low density lipoproteins*.
Low density lipoprotein: A type of molecule found in the bloodstream that has a lower *protein-*to-*lipid* ratio compared with high density lipoproteins.
Lymphoid tissues: The tissues in which *B cells* and *T cells* develop and congregate to initiate an immune response, including the bone marrow, *thymus*, lymph nodes, spleen, and tonsils.
Macrophage: An immune cell that has left the bloodstream and resides in the tissues and which is responsible for consuming foreign bodies such as bacteria. Macrophages also process and present *antigen* to *T cells* and secrete *cytokines* and *complement proteins*.
Mean corpuscular volume (MCV): A measure of the average size of a sample of red blood cells.
Messenger RNA (mRNA): A type of *RNA* molecule that carries the information from a *gene* and serves as a template for the production of *proteins*.
Microcirculation: The blood flow throughout the system of small blood vessels in the body.
Mitochondria: Cell components that generate energy.
Motility: Spontaneous and involuntary movement (e.g., of muscles involved in the gastrointestinal tract).
Mucosa: A thin tissue layer that lines cavities or canals of the body that open to the outside (e.g., regions of the gastrointestinal tract or the nose). The mucosa secretes mucus and absorbs water, salts, and other substances.
Mucosal barrier: The ability of the *epithelium* to prevent the transfer of substances from the gastric or intestinal cavity into the *mucosa*.
Monocyte: A *phagocyte* that originates in the bone marrow and circulates in the bloodstream. Monocytes also enter the tissues, where they mature into *macrophages*.
Natural killer (NK) cell: A type of *white blood cell* that kills tumor cells and virus-infected cells.
Necrosis: Death of one or more cells or of a portion of tissue or organ resulting from irreversible damage.
Nephron: The functional unit of the kidney, each consisting of a *glomerulus* surrounded by a capsule that connects to a long, looping tubule system.
Neuron: A nerve cell.
Neurotransmitter: A chemical released by *neurons* that causes a reaction in nerve, muscle, or gland cells.
Neutropenia: A blood condition in which the number of *neutrophils* is lower than normal.
Neutrophil: A type of *white blood cell* that performs phagocytic and degradative functions similar to those of *macrophages*.
Nucleotide: The building block of *DNA* or *RNA*. Specific strings of DNA nucleotides make up *genes*.
Osmosis: The movement of water across a membrane from the more dilute side to the more concentrated side.
Osmolality: A measurement of concentration of *solutes* in a solution.
Oxidation: A type of chemical reaction usually involving loss of hydrogen.
Pancytopenia: A blood condition in which the numbers of red blood cells, *white blood cells*, and *platelets* are lower than normal.
Parotid glands: The largest salivary glands, which lie just below and in front of the ears.
Permeability: The degree to which a membrane allows various molecules to pass through it.
Phagocyte: A *white blood cell* capable of ingesting (i.e., phagocytosing) foreign particles and microorganisms. Phagocytes include *monocytes, macrophages*, and *neutrophils*.
Phenotype: The observable properties, traits, or physical appearance of an organism resulting from the interaction of the *genotype* with environmental factors.
Plasma: The watery portion of the blood, in which the blood cells are suspended; the plasma contains minerals, nutrients, regulatory substances, gases, and *proteins*.
Plasma cell: A *B cell* that has been activated by *antigen* to produce and secrete large amounts of *antibodies*.
Plasmin: An *enzyme* that digests the *protein fibrin* in the dissolution of blood clots; normally present in the blood in the form of its inactive precursor, plasminogen.
Plasminogen activators: A *protein* that activates the precursor plasminogen to its active form, *plasmin*, to initiate the dissolution of a blood clot.
Platelets: Disk-shaped components of blood that aggregate to stop bleeding during the clotting process.
Polymorphism: For a specific *gene*, the presence of two or more gene variants (i.e., *alleles*) in a population.
Protein: The product of the genetic information encoded in a *gene*. Proteins are made up of chains of *amino acids*, whose order and synthesis are dictated by the gene’s *nucleotide* sequence. *Enzymes* are one type of protein.
Receptor: A *protein* usually found on the surface of a *neuron* or other cell that recognizes and binds to *neurotransmitters* or other chemical messengers.
RNA: The abbreviation for ribonucleic acid, a *DNA*-like molecule that plays a role in using genetic information (i.e., DNA) to produce *proteins*.
Sarcoplasmic reticulum: A system of interconnected tubules within heart muscle cells with functions related to the transmission of nervous excitation to the contractile parts of the heart muscle.
Solute: The substance dissolved in solution (e.g., salt is the solute in the solution known as salt water).
Stroke: Any condition during which the blood supply to the brain or regions of the brain is suddenly interrupted.
Sympathetic nervous system: The division of the nervous system that coordinates the body’s response to stress.
Synapse: A microscopic gap separating adjacent *neurons* where *neurotransmitters* and *receptors* cluster.
Temporal lobe: The region of the *cerebral cortex* forming part of the sides and bottom of the brain. This region is involved in sensory processing, language functions, and emotions.
Thalamus: A mass of gray matter that forms the lateral walls of the *diencephalon* and which is involved in the transmission and integration of certain sensations.
Thiamine: Vitamin B_1_; essential for the health of the cardiovascular and nervous systems.
Thrombocytopenia: A blood condition in which the number of *platelets* is below normal.
Thromboxane A_2_: A compound that strongly stimulates *platelet* aggregation and activation in the process of blood clot formation.
Thrombus: A clot of blood within the heart or blood vessels. If a detached thrombus is carried in the blood and lodges at a later point, it is called an embolus.
Thymus: A *lymphoid* organ, located near the base of the neck, where *T cells* mature.
T lymphocyte (T cell): A type of *white blood cell* that originates in the bone marrow and matures in the *thymus*. T cells are activated by contact with *antigen* to mount a *cell-mediated immune response* and secrete important *cytokines*.
Transforming growth factor beta (TGF-β): A *cytokine* that has multiple functions, including regulation of other cytokines. It is produced by *platelets*, bone cells, and many other cell types.
Triglyceride: A *lipid* or neutral fat that serves as a metabolic energy source.
Tumor necrosis factor (TNF): A *cytokine* produced by *macrophages* that has anticancer effects.
Up-regulation: An increase in the number or sensitivity of *receptors* as a regulatory mechanism to compensate for decreased activation of the receptors.
Ventricular fibrillation: Rapid and uncoordinated contraction of the lower chambers of the heart, most often resulting from restriction or interruption of the heart muscle’s blood supply. The heart then ceases to pump blood.
Very low density lipoprotein (VLDL): A type of molecule formed primarily in the liver to transport *cholesterol* and *triglycerides* in the blood to body tissues.
White blood cell: Immune cells that make up the first line of defense against infection and toxic agents; also called *leukocytes*.

**Figure:**
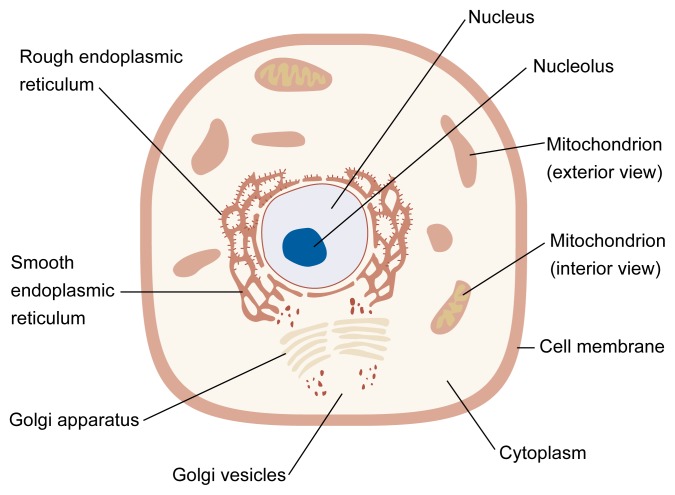
Schematic illustration of a typical cell. The nucleus contains the cell’s DNA and controls cell reproduction; the nucleolus is a substructure of the nucleus that contains large quantities of RNA and plays a role in the cell’s synthesis of proteins. Filling the cell is cytoplasm, a substance containing proteins, electrolytes, glucose, and tiny structures called organelles. One type of organelle, the mitochondrion, converts nutrients to chemicals that provide energy for the cell. The rough endoplasmic reticulum—so called because of the RNA particles attached to its surface—synthesizes proteins, and the smooth endoplasmic reticulum synthesizes lipids for the cell’s use. As these molecules are formed, some of them break away from the endoplasmic reticulum and move into the Golgi apparatus. After additional processing, the new substances are released into the cytoplasm in small globules called vesicles, where they may be used within the cell (e.g., for cell membrane replenishment) or secreted through the cell membrane for use outside the cell. In liver cells, the endoplasmic reticulum is also the locus for the microsomal enzyme oxidizing system (MEOS). The MEOS uses the cytochrome P3450 2E1 to convert alcohol to acetaldehyde.

